# Inhibition of Cdk2 kinase activity selectively targets the CD44^+^/CD24^−/Low^ stem-like subpopulation and restores chemosensitivity of SUM149PT triple-negative breast cancer cells

**DOI:** 10.3892/ijo.2014.2523

**Published:** 2014-06-25

**Authors:** MATEUSZ OPYRCHAL, JEFFREY L. SALISBURY, IANKO IANKOV, MATHEW P. GOETZ, JAMES McCUBREY, MARIO W. GAMBINO, LORENZO MALATINO, GIUSEPPE PUCCIA, JAMES N. INGLE, EVANTHIA GALANIS, ANTONINO B. D’ASSORO

**Affiliations:** 1 Department of Medical Oncology, Mayo Clinic College of Medicine, Rochester, MN, USA; 2 Department of Biochemistry and Molecular Biology, Mayo Clinic College of Medicine, Rochester, MN, USA; 3 Department of Molecular Medicine, Mayo Clinic College of Medicine, Rochester, MN, USA; 4 Department of Microbiology and Immunology, East Carolina University, Greenville, NC, USA; 5 Department of Internal Medicine, University of Catania, Catania, Italy

**Keywords:** breast cancer, metastases, cell cycle, cancer stem cells, cyclin E, centrosome amplification, chromosomal instability

## Abstract

Inflammatory breast cancer (IBC) is an angioinvasive and most aggressive type of advanced breast cancer characterized by rapid proliferation, chemoresistance, early metastatic development and poor prognosis. IBC tumors display a triple-negative breast cancer (TNBC) phenotype characterized by centrosome amplification, high grade of chromosomal instability (CIN) and low levels of expression of estrogen receptor α (ERα), progesterone receptor (PR) and HER-2 tyrosine kinase receptor. Since the TNBC cells lack these receptors necessary to promote tumor growth, common treatments such as endocrine therapy and molecular targeting of HER-2 receptor are ineffective for this subtype of breast cancer. To date, not a single targeted therapy has been approved for non-inflammatory and inflammatory TNBC tumors and combination of conventional cytotoxic chemotherapeutic agents remains the standard therapy. IBC tumors generally display activation of epithelial to mesenchymal transition (EMT) that is functionally linked to a CD44^+^/CD24^−/Low^ stem-like phenotype. Development of EMT and consequent activation of stemness programming is responsible for invasion, tumor self-renewal and drug resistance leading to breast cancer progression, distant metastases and poor prognosis. In this study, we employed the luminal ER^+^ MCF-7 and the IBC SUM149PT breast cancer cell lines to establish the extent to which high grade of CIN and chemoresistance were mechanistically linked to the enrichment of CD44^+^/CD24^low/−^ CSCs. Here, we demonstrate that SUM149PT cells displayed higher CIN than MCF-7 cells characterized by higher percentage of structural and numerical chromosomal aberrations. Moreover, centrosome amplification, cyclin E overexpression and phosphorylation of retinoblastoma (Rb) were restricted to the stem-like CD44^+^/CD24^−/Low^ subpopulation isolated from SUM149PT cells. Significantly, CD44^+^/CD24^−/Low^ CSCs displayed resistance to conventional chemotherapy but higher sensitivity to SU9516, a specific cyclin-dependent kinase 2 (Cdk2) inhibitor, demonstrating that aberrant activation of cyclin E/Cdk2 oncogenic signaling is essential for the maintenance and expansion of CD44^+^/CD24^−/Low^ CSC subpopulation in IBC. In conclusion, our findings propose a novel therapeutic approach to restore chemosensitivity and delay recurrence of IBC tumors based on the combination of conventional chemotherapy with small molecule inhibitors of the Cdk2 cell cycle kinase.

## Introduction

Inflammatory breast cancer (IBC) is an angioinvasive form of breast cancer in which cancer cells block the lymphatic vessels in the skin covering the breast and it is associated with a high incidence of early nodal and systemic metastases (1). This type of breast cancer is called ‘inflammatory’ because the breast often looks swollen and red, resembling an inflammatory condition (2). Although IBC accounts for only 2–5% of all breast cancers, IBC tumors show a poor prognosis with 40% five-years survival rate versus 87% for all breast cancers combined, making IBC a priority area for the development of new therapeutic strategies (3). IBC tumors display a triple negative breast cancer (TNBC) phenotype characterized by high chromosomal instability (CIN) and low levels of expression of estrogen receptor α (ERα), progesterone receptor (PR) and HER-2 tyrosine kinase receptor (4). Since the TNBC cells lack these receptors necessary to promote tumor growth, common treatments such as endocrine therapy and molecular targeting of HER-2 receptor are ineffective for this subtype of breast cancer (5). Employing conventional chemotherapy to treat TNBC tumors is still an effective option because TNBC tumors may respond even better to chemotherapeutic agents in the earlier stages than many other forms of breast cancer (6). Unfortunately, TNBC tumors will likely develop chemoresistance leading to tumor progression, metastatic spreading to distant organs and poor outcome (7).

Germline mutations in the BRCA1 and BRCA2 breast cancer susceptibility genes have been associated with up to 15% of TNBC, and TNBC accounts for 70% of breast tumors arising in BRCA1 mutation carriers and 16–23% of breast tumors in BRCA2 carriers (8). Because BRCA1 and BRCA2 tumor suppressor genes play a key role in the control of genomic stability through regulation of DNA repair and centrosome duplication (9–11), these findings explain the causal role of BRCA1 and BRCA2 mutations in the development of high CIN commonly observed in TNBC tumors. Moreover, TNBC tumors frequently overexpress cyclin E, a late G1 cyclin that binds and activates cyclin-dependent kinase 2 (Cdk2) leading to phosphorylation and inactivation of retinoblastoma (Rb) essential for the G1-S phase transition of the cell cycle (12,13). Importantly, aberrant activation of cyclin E/Cdk2 complex plays a key role in the development of centrosome amplification, CIN and breast cancer progression and may represent an attractive ‘druggable oncogenic signaling’ for the treatment of TNBC tumors lacking molecular targeted therapy (14–16). Recent studies have demonstrated that breast carcinomas are composed of a singular subpopulation of cells harboring stem-like properties termed cancer stem cells (CSCs) (17). CSCs show activation of epithelial to mesenchymal transition (EMT) programming and acquire a basal-like CD44^+^/CD24^low/−^ phenotype with increased capacity for self-renewal, invasion, drug resistance and tumor progression (18–20). The discovery of CD44^+^/CD24^low/−^ CSCs has generated excitement because this subpopulation of cancer cells may represent a source of therapeutic failure to anticancer drugs. Significantly, it has been demonstrated that treatment of breast tumors with conventional chemotherapeutic agents enriched the CD44^+^/CD24^low/−^ subpopulation conferring resistance to the initial treatment and leading to the development of distant metastases (21). In agreement with these findings, TNBC tumors generally display a basal-like CD44^+^/CD24^low/−^ phenotype that clarifies their resistance to conventional chemotherapy responsible for high risk of recurrence and poor prognosis (22).

In this study we employed the luminal ER^+^ MCF-7 and the IBC SUM149PT breast cancer cells to establish the extent to which high grade of CIN and chemoresistance was mechanistically linked to the enrichment of CD44^+^/CD24^low/−^ CSCs. We demonstrate that overexpression of cyclin E is restricted to the stem-like CD44^+^/CD24^low/−^ subpopulation and is functionally linked to phosphorylation of retinoblastoma (Rb), centrosome amplification and genomic instability. Significantly, CD44^+^/CD24^low/−^ CSCs displayed higher sensitivity to a specific Cdk2 inhibitor than the bulk SUM149PT cells indicating that aberrant activation of cyclin E/Cdk2 oncogenic signaling is essential for the maintenance and expansion of CD44^+^/CD24^low/−^ CSCs subpopulation in IBC. In conclusion our findings highlight a novel therapeutic approach based on the combination of conventional chemotherapy with small molecule inhibitors of the Cdk2 cell cycle kinase to treat chemoresistant IBC tumors.

## Materials and methods

### Human breast cancer cell lines

The human breast cancer cell line MCF-7 was obtained from ATCC (Manassas, VA, USA), normal human mammary epithelial cells HMEC and IBC SUM149PT cancer cells were kindly provided by Dr Lingle and Dr Couch’s laboratories, respectively (Mayo Clinic, Rochester, MN). All cell lines were maintained in EMEM medium containing 5 mM glutamine, 1% penicillin/streptomycin, 20 microgram insulin/ml and 10% FBS at 37°C in 5% CO_2_ atmosphere.

### Cytogenetic and SKY analysis

Cell harvest and metaphase slide preparation for routine cytogenetic and spectral karyotyping (SKY) analysis were performed as previously described (23–25). Hybridization, wash and detection of the human SKYPaint^®^ probe (Applied Spectral Imaging; Vista, CA) were performed as recommended by the manufacturer. Image acquisition and spectral analysis of metaphase cells were achieved by using the SD200 SpectraCube™ Spectral Imaging system (Applied Spectral Imaging) mounted on a Zeiss Axioplan2 microscope (Carl Zeiss MicroImaging, Inc., Thornwood, NY). Images were analyzed using HiSKY analysis software (Applied Spectral Imaging).

### FACS analysis

CD44 and CD24 antibodies (Abcam, Cambridge, MA, USA) were employed to identify and isolate CD44^+^/CD24^−^ breast cancer initiating cells by FACS sorting analysis as previously described (20).

### Immunoblot and immunofluorescence assays

Immunoblot and immunofluorescence studies were performed as previously described (27). Antibodies employed for the immunoblot and immunofluorescence assays were: pericentrin (Abcam), cyclin E (Santa Cruz Biotechnology, Santa Cruz, CA), P~Rb and β-actin (Sigma, St. Louis, MO). Results are derived from three independent experiments.

### Chemoresistance studies

For chemoresistance studies, 5×10^4^ cancer cells were plated in 6-well costar plates and cultured in complete EMEM medium for 48 h. Following 48-h incubation, cancer cells were treated with 1 μM methotrexate, 0.5 μM paclitaxel alone or in combination with 1 μM SU9516 (Sigma) for additional 72 h. Cytotoxicity of conventional chemotherapy alone and in combination with SU9516 was tested by immunofluorescence employing a cleaved-PARP antibody (Cell Signaling Technology, Danvers, MA) as a marker of apoptosis. Results are derived from three independent experiments.

## Results

**Figure 1:**
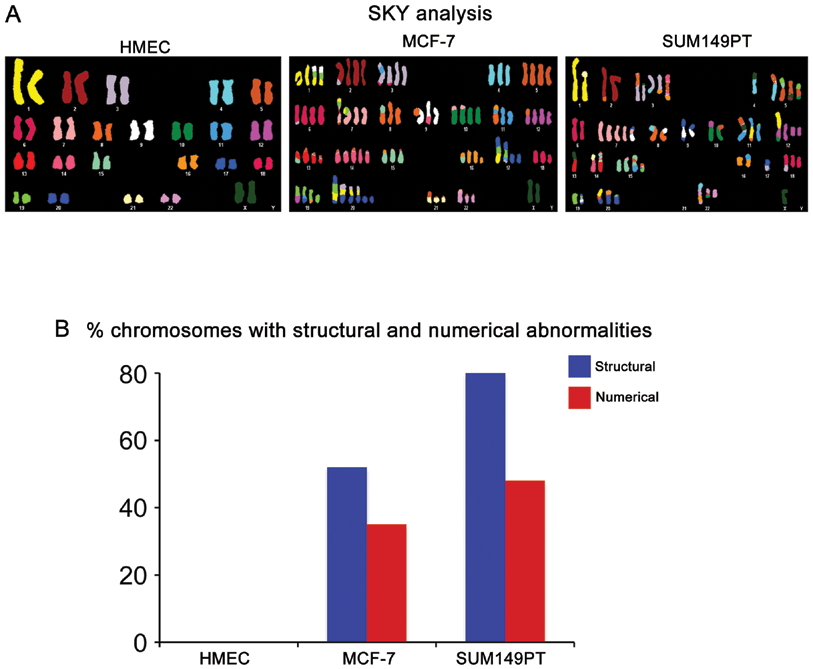
SKY analysis of human breast cancer cell lines. (A) Representative structural and numerical chromosomal abnormalities identified through SKY analysis in MCF-7 and SUM149PT cancer cells. Normal HMEC cells were used as control. (B) Graph showing the percentage of total structural and numerical chromosomal abnormalities identified in human breast cancer cells through cytogenetic analysis and SKY.

In view of the fact that IBC tumors commonly display higher grade of CIN compared to luminal ERα^+^ breast tumors, we employed luminal ERα^+^ MCF-7 and IBC SUM149PT cancer cell lines to investigate their level of chromosomal abnormalities; normal mammary epithelial HMEC cells were used as control. The SUM149PT cancer cell line was isolated from an IBC tumor and carries the 2288delT mutation that is linked to loss of *BRCA1* function and represents an excellent preclinical model to study the molecular mechanisms responsible for the development of chemoresistance and tumor progression in IBC tumors (23). We performed an integrative karyotypic analysis of HMEC, MCF-7 and SUM149PT cells employing the spectral karyotyping (SKY) technology and routine cytogenetic analysis (Fig. 1A). Breast cancer cell lines were harvested and metaphase spreads for cytogenetic and SKY analyses were prepared as previously described (24). Comparison of the three cell lines showed that while MCF-7 and SUM149PT cancer cells displayed a variety of chromosomal abnormalities, HMEC cells exhibited a normal diploid karyotype. Significantly, SUM149PT cells displayed a higher rate of CIN characterized by higher percentage of structural and numerical chromosomal abnormalities compared to MCF-7 cells (Fig. 1B).

**Figure 2:**
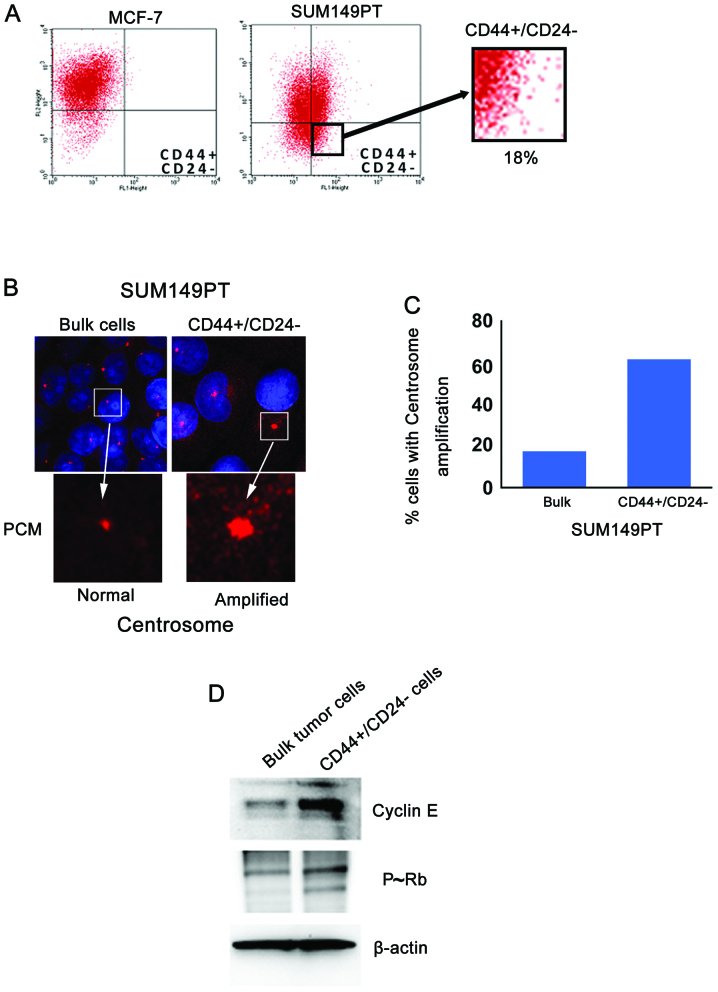
Isolation and molecular characterization of CD44^+^/CD24^−/Low^ CSCs. (A) FACS analysis showing the percentage of CD44^+^/CD24^−/Low^ CSCs in MCF-7 and SUM149PT cancer cells. (B) Immunofluorescence of centrosomes in SUM149PT cancer cells and CD44^+^/CD24^−/Low^ CSCs. Centrosomes were labeled in red with pericentrin and nuclei were labeled in blue with DAPI (C). Graph showing the percentage of breast cancer cells harboring centrosome amplification. (D) Immunoblot showing cyclin E and P~Rb expression in SUM149PT cancer cells and CD44^+^/CD24^−/Low^ CSCs. β-actin was employed as loading control. Experiments were performed in triplicate.

To establish the extent to which the higher level of CIN observed in the SUM149PT cancer cells was functionally linked to the presence of a stem-like CD44^+^/CD24^−/Low^ subpopulation, FACS analysis was performed on MCF-7 and SUM149PT cancer cells to analyze the percentage of cells displaying a CD44^+^/CD24^−/Low^ phenotype. While MCF-7 cells showed mainly a luminal CD44^−^/CD24^+^ phenotype, 18% of SUM149PT cells exhibited a stem-like CD44^+^/CD24^−/Low^ phenotype (Fig. 2A). Because CIN in breast cancer is mechanistically linked to development of centrosome abnormalities, we analyzed the grade of centrosome amplification in bulk SUM149PT cancer cells versus the stem-like CD44^+^/CD24^−/Low^ subpopulation isolated by FACS sorting assay. Centrosome amplification in cancer cells was examined by labeling the centrosome size with pericentrin, an oncoprotein that is localized in the pericentriolar material (25). Significantly, the CD44^+^/CD24^−/Low^ subpopulation revealed higher centrosome amplification compared to bulk SUM149PT cancer cells (Fig. 2B and C), suggesting that the higher degree of CIN observed in SUM149PT cancer cells was functionally linked to the genesis of CD44^+^/CD24^−/Low^ CSCs harboring amplified centrosomes.

Next we investigated the molecular mechanisms responsible for the development of centrosome abnormalities in CD44^+^/CD24^−/Low^ CSCs. Because the oncoprotein cyclin E plays a key role in the development of centrosome abnormalities and is frequently overexpressed in TNBC tumors, we investigated the expression and activity of cyclin E in SUM149PT cells versus the CD44^+^/CD24^−/Low^ subpopulation isolated by FACS sorting assay. Importantly, immunoblot analysis showed that cyclin E was overexpressed in the CD44^+^/CD24^−/Low^ subpopulation compared to bulk SUM149PT cells (Fig. 2D). To investigate whether cyclin E overexpression was functionally linked to aberrant Cdk2 kinase activity, we analyzed the phosphorylation status of Rb tumor suppressor that is a downstream target of cyclin E/Cdk2 oncogenic signaling and its inactivation leads to centrosome aberrations (26). Higher phosphorylation and consequent inactivation of Rb was observed in the CD44^+^/CD24^−/Low^ subpopulation compared to bulk SUM149PT cancer cells (Fig. 2D). Taken together these findings reveal that centrosome amplification was mechanistically linked to aberrant cyclin E/Cdk2 activity and consequent loss of Rb function in CD44^+^/CD24^−/Low^ CSCs.

**Figure 3:**
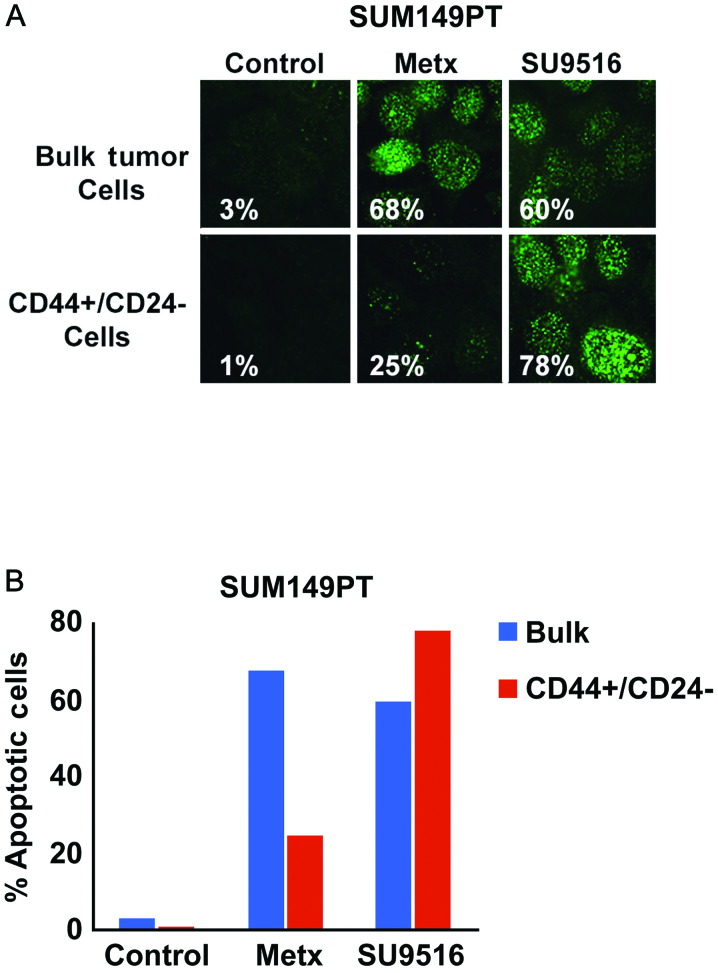
Treatment of SUM149PT cancer cells and CD44^+^/CD24^−/Low^ CSCs with chemotherapeutic agents. (A) Immunofluorescence analysis showing activation of apoptosis in SUM149PT cancer cells and CD44^+^/CD24^−/Low^ CSCs treated with 1 μM methotrexate or 1 μM SU9516. Cleaved PARP indicating activation of apoptosis was labeled in green. (B) Graph showing the percentage of apoptotic SUM149PT cancer cells and CD44^+^/CD24^−/Low^ CSCs following treatment with 1 μM methotrexate or 1 μM SU9516. Experiments were performed in triplicate.

**Figure 4:**
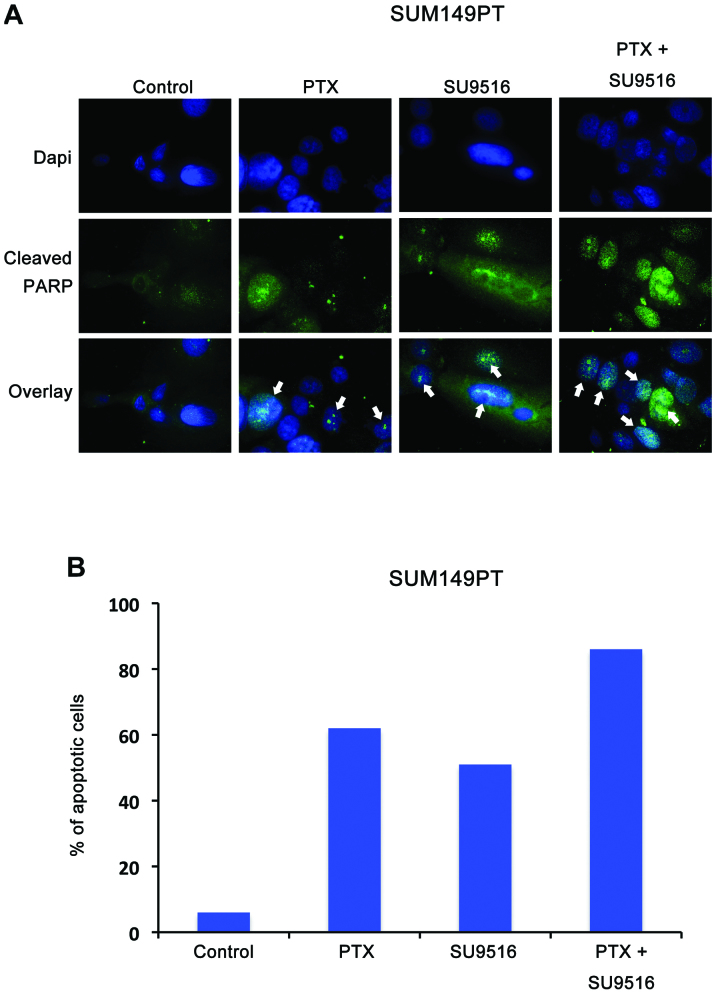
Treatment of SUM149PT cancer cells with paclitaxel and SU9516. (A) Immunofluorescence analysis showing activation of apoptosis in SUM149PT cancer cells treated with 0.5 μM paclitaxel alone and/or 1 μM SU9516. Cleaved PARP indicating activation of apoptosis was labeled in green and nuclei were labeled in blue with DAPI. (B) Graph showing the percentage of apoptotic SUM149PT cancer cells following treatment with 0.5 μM paclitaxel alone and/or 1 μM SU9516. Experiments were performed in triplicate.

Because TNBC tumors display a poor prognosis that is associated to higher chemoresistance and stemness properties compared to luminal breast tumors, we investigated the extent to which inhibition of Cdk2 kinase activity selectively targeted the stem-like CD44^+^/CD24^−/Low^ subpopulation and restored chemosensitivity of SUM149PT cancer cells. Bulk SUM149PT and CD44^+^/CD24^−/Low^ cancer cells were treated with the antimetabolite methotrexate (a conventional chemotherapeutic drug) or SU9516 (a small molecule inhibitor of Cdk2 activity) and cytotoxicity was quantified by analyzing the percentage of cells harboring cleaved PARP as a marker of apoptosis. Significantly, CD44^+^/CD24^−/Low^ CSCs displayed a higher resistance to methotrexate while they where highly sensitive to the Cdk2 inhibitor SU9516 compared to bulk SUM149PT cancer cells (Fig. 3). Finally, we tested *in vitro* the translational therapeutic relevance of combining conventional anticancer agents with small molecule inhibitors of Cdk2 activity to restore chemosensitivity of TNBC tumors. SUM149PT cells were treated with paclitaxel, a taxane commonly employed in the treatment of TNBC tumors, alone or in combination with SU9516. Notably, our study revealed that combination of paclitaxel with SU9516 induced a stronger cytotoxic activity characterized by the majority of SUM149PT cells undergoing apoptosis compared to treatment with paclitaxel or SU9516 alone (Fig. 4).

## Discussion

IBC tumors represent a rare and very aggressive subtype of breast carcinomas that display a TNBC phenotype defined as the absence of staining for ERα, PR and HER-2 receptors (1–3). TNBC tumors are insensitive to some of the most effective targeted therapies available for breast cancer treatment including endocrine therapies such as tamoxifen, aromatase inhibitors or fulvestrant and HER-2-directed therapy such as trastuzumab (27). To date, not a single targeted therapy has been approved for early-stage TNBC tumors and combination of conventional cytotoxic chemotherapeutic agents administered in a dose-dense or metronomic schedule remains the standard therapy (28). Significantly, a prospective analysis of 1,118 patients who received neoadjuvant chemotherapy at a single institution, of whom 255 (23%) had TNBC, found that patients with TNBC tumors had higher pathologic complete response (pCR) rates compared with non-TNBC patients (29). Because pCR is functionally linked to improved long-term outcomes, it is imperative to develop innovative targeted therapeutic strategies aimed to increase pCR rates with consequent benefits on the disease-free and overall survival of TNBC patients. Moreover, the use of anthracyclines (such as doxorubicin and epirubicin) and taxanes (such as paclitaxel and docetaxel) as chemotherapeutic agents for IBC tumors have been shown to improve outcomes (30). Although IBC tumors respond well initially to conventional chemotherapy, they often develop chemoresistance leading to tumor progression and poor outcome (31).

Development of EMT and consequent activation of stemness programming is responsible for invasion, tumor self-renewal and drug resistance leading to breast cancer progression, distant metastases and poor prognosis (32). For this reason the cancer stem cell-like phenotype commonly observed in IBC tumors may contribute to their aggressive nature but also may offer itself novel therapeutic strategies to the selective targeting of CD44^+^/CD24^−^ CSCs from bulk tumors. Abrogation of BRCA1 and BRCA2 genes function can in part explain the high grade of CIN observed in inflammatory and non-inflammatory TNBC tumors resulting from centrosome amplification and impairment of the DNA repair machinery (10). Breast tumors carrying BRCA1 mutations are considered to be sensitive to inhibitors of poly (ADP-ribose) polymerases (PARPs) that are nuclear enzymes implicated in cellular responses to DNA injury provoked by genotoxic stress (33). PARP-1, the best characterized member of the PARP family, is essential to the repair of DNA single-strand breaks via the base excision repair pathway (34). Inhibitors of PARP-1 have been shown to enhance the cytotoxic effects of ionizing radiation and DNA-damaging chemotherapy agents, such as the methylating agents and topoisomerase II inhibitors (35). Moreover, recent studies suggests that PARP inhibitors could be used not only as chemo/radiotherapy sensitizers, but also as single agents to selectively kill cancers defective in DNA repair, specifically cancers with mutations in the breast cancer-associated genes BRCA1 and BRCA2 (36). Although clinical trials indicate that PARP-inhibitors have emerged as a promising new class of antineoplastic agents, significant numbers of TNBC tumors still recur (37). TNBC tumors recurrence following treatment with PARP-inhibitors can be explained by recent studies demonstrating that PARP-inhibitors eliminate the bulk of tumor cells, but they have limited ability to eliminate CD44^+^/CD24^−^ CSCs (38). To date no preclinical study has demonstrated the efficacy of PARP-inhibitors in selective targeting of CD44^+^/CD24^−^ CSCs in IBC tumors. For this reason the discovery of oncogenic signalings responsible for the maintenance and expansion of CD44^+^/CD24^−^ CSCs is crucial for the development of innovative-targeted therapies aimed to drastically reduce the recurrence and poor outcome of IBC tumors.

The findings presented in this study demonstrate that IBC SUM149PT cancer cells exhibit higher CIN based on structural and numerical chromosome abnormalities than luminal ERα^+^ MCF-7 cancer cells. Moreover, we established that only SUM149PT cancer cells contain a CD44^+^/CD24^−/Low^ CSCs subpopulation displaying centrosome amplification that was functionally linked to cyclin E overexpression and Rb phosphorylation. Several studies have shown that elevated levels of cyclin E induce aberrant activation of Cdk2 kinase leading to Rb phosphorylation and inactivation with deleterious consequences on the control of centrosome duplication and CIN (14–16). Significantly, cyclin E is overexpressed in aggressive breast tumors and it has been associated with CIN, development of distant metastases and poor outcome (39). Concurring with our findings, it was previously demonstrated that SUM149PT cancer cells display very high levels of cyclin E expression for the duration of the cell cycle which is in contrast to cyclin E degradation observed in the mid to late S phase of normal cells. In addition, comparative genomic hybridization indicated that SUM149PT cells exhibit many chromosome copy number alterations, which may reflect prior or ongoing chromosome instability driven by cyclin E activity (40). Because we have demonstrated that cyclin E overexpression is restricted to the subpopulation of CD44^+^/CD24^−^ CSCs isolated from SUM149PT cells, we treated bulk SUM149PT cells and CD44^+^/CD24^−^ CSCs with a conventional anticancer drug (methotrexate) and a specific Cdk2 inhibitor (SU9516) to abrogate the cyclin E/Cdk2 oncogenic signaling. CSCs showed higher resistance to methotrexate than bulk SUM149PT cells, nonetheless CSCs were more sensitive to the cytotoxic effects of SU9516 indicating that molecular inhibition of Cdk2 kinase activity selectively targeted CSCs in IBC. Finally, we showed that combination of paclitaxel with SU9516 *in vitro* induces a stronger cytotoxic effect characterized by activation of apoptosis in SUM149PT cells. Importantly, the preclinical relevance of our findings is justified by recent studies demonstrating that administration of cyclin E siRNA *in vivo* inhibited breast tumor growth in nude mice. Moreover, the authors demonstrate that cyclin E siRNA synergistically enhanced the cell killing effects of chemotherapeutic agents *in vitro* and this combination greatly suppressed the tumor growth in nude mice. In conclusion our study demonstrate for the first time that aberrant activation of cyclin E/Cdk2 oncogenic signaling is restricted in CSCs derived from IBC cells and combination of conventional chemotherapy with small molecule inhibitors of Cdk2 kinase activity may represent a novel targeted therapeutic approach to treat aggressive IBC tumors with consequent benefits on the disease-free and overall survival of patients.

## References

[1] Fernandez SV, Robertson FM, Pei J, Aburto-Chumpitaz L, Mu Z, Chu K, Alpaugh RK, Huang Y, Cao Y, Ye Z, Cai KQ, Boley KM, Klein-Szanto AJ, Devarajan K, Addya S, Cristofanilli M (2013). Inflammatory breast cancer (IBC): clues for targeted therapies. Breast Cancer Res Treat.

[2] Yasumura K, Ogawa K, Ishikawa H, Takeshita T, Nakagawa Y, Osamura RY (1997). Inflammatory carcinoma of the breast: characteristic findings of MR imaging. Breast Cancer.

[3] Yamauchi H, Woodward WA, Valero V, Alvarez RH, Lucci A, Buchholz TA, Iwamoto T, Krishnamurthy S, Yang W, Reuben JM, Hortobágyi GN, Ueno NT (2012). Inflammatory breast cancer: what we know and what we need to learn. Oncologist.

[4] Liedtke C, Bernemann C, Kiesel L, Rody A (2013). Genomic profiling in triple-negative breast cancer. Breast Care (Basel).

[5] Qiu M, Peng Q, Jiang I, Carroll C, Han G, Rymer I, Lippincott J, Zachwieja J, Gajiwala K, Kraynov E, Thibault S, Stone D, Gao Y, Sofia S, Gallo J, Li G, Yang J, Li K, Wei P (2013). Specific inhibition of Notch1 signaling enhances the antitumor efficacy of chemotherapy in triple negative breast cancer through reduction of cancer stem cells. Cancer Lett.

[6] Cortazar P, Zhang L, Untch M, Mehta K, Costantino JP, Wolmark N, Bonnefoi H, Cameron D, Gianni L, Valagussa P, Swain SM, Prowell T, Loibl S, Wickerham DL, Bogaerts J, Baselga J, Perou C, Blumenthal G, Blohmer J, Mamounas EP, Bergh J, Semiglazov V, Justice R, Eidtmann H, Paik S, Piccart M, Sridhara R, Fasching PA, Slaets L, Tang S, Gerber B, Geyer CE, Pazdur R, Ditsch N, Rastogi P, Eiermann W, von Minckwitz G (2014). Pathological complete response and long-term clinical benefit in breast cancer: the CTNeoBC pooled analysis. Lancet.

[7] Crown J, O’Shaughnessy J, Gullo G (2012). Emerging targeted therapies in triple-negative breast cancer. Ann Oncol.

[8] Greenup R, Buchanan A, Lorizio W, Rhoads K, Chan S, Leedom T, King R, McLennan J, Crawford B, Kelly Marcom P, Shelley Hwang E (2013). Prevalence of BRCA mutations among women with triple-negative breast cancer (TNBC) in a genetic counseling cohort. Ann Surg Oncol.

[9] Venkitaraman AR (2002). Cancer susceptibility and the functions of BRCA1 and BRCA2. Cell.

[10] Farrugia DJ, Agarwal MK, Pankratz VS, Deffenbaugh AM, Pruss D, Frye C, Wadum L, Johnson K, Mentlick J, Tavtigian SV, Goldgar DE, Couch FJ (2008). Functional assays for classification of BRCA2 variants of uncertain significance. Cancer Res.

[11] Kais Z, Chiba N, Ishioka C, Parvin JD (2012). Functional differences among BRCA1 missense mutations in the control of centrosome duplication. Oncogene.

[12] Kolupaeva V, Basilico C (2012). Overexpression of cyclin E/CDK2 complexes overcomes FGF-induced cell cycle arrest in the presence of hypophosphorylated Rb proteins. Cell Cycle.

[13] Reed SI (1997). Control of the G1/S transition. Cancer Surv.

[14] D’Assoro AB, Lingle WL, Salisbury JL (2002). Centrosome amplification and the development of cancer. Oncogene.

[15] D’Assoro AB, Busby R, Suino K, Delva E, Almodovar- Mercado GJ, Johnson H, Folk C, Farrugia DJ, Vasile V, Stivala F, Salisbury JL (2004). Genotoxic stress leads to centrosome amplification in breast cancer cell lines that have an inactive G1/S cell cycle checkpoint. Oncogene.

[16] Hanashiro K, Kanai M, Geng Y, Sicinski P, Fukasawa K (2008). Roles of cyclins A and E in induction of centrosome amplification in p53-compromised cells. Oncogene.

[17] Mani SA, Guo W, Liao MJ, Eaton EN, Ayyanan A, Zhou AY, Brooks M, Reinhard F, Zhang CC, Shipitsin M, Campbell LL, Polyak K, Brisken C, Yang J, Weinberg RA (2008). The epithelial mesenchymal transition generates cells with properties of stem cells. Cell.

[18] Hwang-Verslues WW, Kuo WH, Chang PH, Pan CC, Wang HH, Tsai ST, Jeng YM, Shew JY, Kung JT, Chen CH, Lee EY, Chang KJ, Lee WH (2009). Multiple lineages of human breast cancer stem/progenitor cells identified by profiling with stem cell markers. PLoS One.

[19] Shipitsin M, Campbell LL, Argani P, Weremowicz S, Bloushtain-Qimron N, Yao J, Nikolskaya T, Serebryiskaya T, Beroukhim R, Hu M, Halushka MK, Sukumar S, Parker LM, Anderson KS, Harris LN, Garber JE, Richardson AL, Schnitt SJ, Nikolsky Y, Gelman RS, Polyak K (2007). Molecular definition of breast tumor heterogeneity. Cancer Cell.

[20] D’Assoro AB, Liu T, Quatraro C, Amato A, Opyrchal M, Leontovich A, Ikeda Y, Ohmine S, Lingle W, Suman V, Ecsedy J, Iankov I, Di Leonardo A, Ayers-Inglers J, Degnim A, Billadeau D, McCubrey J, Ingle J, Salisbury JL, Galanis E (2014). The mitotic kinase Aurora-A promotes distant metastases by inducing epithelial-to-mesenchymal transition in ERα(+) breast cancer cells. Oncogene.

[21] Lee HE, Kim JH, Kim YJ, Choi SY, Kim SW, Kang E, Chung IY, Kim IA, Kim EJ, Choi Y, Ryu HS, Park SY (2011). An increase in cancer stem cell population after primary systemic therapy is a poor prognostic factor in breast cancer. Br J Cancer.

[22] Arima Y, Hayashi N, Hayashi H, Sasaki M, Kai K, Sugihara E, Abe E, Yoshida A, Mikami S, Nakamura S, Saya H (2012). Loss of p16 expression is associated with the stem cell characteristics of surface markers and therapeutic resistance in estrogen receptor-negative breast cancer. Int J Cancer.

[23] Prat A, Karginova O, Parker JS, Fan C, He X, Bixby L, Harrell JC, Roman E, Adamo B, Troester M, Perou CM (2013). Characterization of cell lines derived from breast cancers and normal mammary tissues for the study of the intrinsic molecular subtypes. Breast Cancer Res Treat.

[24] D’Assoro AB, Leontovich A, Amato A, Ayers-Ringler JR, Quatraro C, Hafner K, Jenkins RB, Libra M, Ingle J, Stivala F, Galanis E, Salisbury JL (2010). Abrogation of p53 function leads to metastatic transcriptome networks that typify tumor progression in human breast cancer xenografts. Int J Oncol.

[25] D’Assoro AB, Barrett SL, Folk C, Negron VC, Boeneman K, Busby R, Whitehead C, Stivala F, Lingle WL, Salisbury JL (2002). Amplified centrosomes in breast cancer: a potential indicator of tumor aggressiveness. Breast Cancer Res Treat.

[26] Iovino F, Lentini L, Amato A, Di Leonardo A (2006). RB acute loss induces centrosome amplification and aneuploidy in murine primary fibroblasts. Mol Cancer.

[27] Mayer IA, Abramson VG, Lehmann BD, Pietenpol JA (2014). New strategies for triple-negative breast cancer - deciphering the heterogeneity. Clin Cancer Res.

[28] Mehta RS (2008). Dose-dense and/or metronomic schedules of specific chemotherapies consolidate the chemosensitivity of triple-negative breast cancer: a step toward reversing triple-negative paradox. J Clin Oncol.

[29] Liedtke C, Mazouni C, Hess KR, André F, Tordai A, Mejia JA, Symmans WF, Gonzalez-Angulo AM, Hennessy B, Green M, Cristofanilli M, Hortobagyi GN, Pusztai L (2008). Response to neoadjuvant therapy and long-term survival in patients with triple-negative breast cancer. J Clin Oncol.

[30] Cristofanilli M, Buzdar AU, Sneige N, Smith T, Wasaff B, Ibrahim N, Booser D, Rivera E, Murray JL, Valero V, Ueno N, Singletary ES, Hunt K, Strom E, McNeese M, Stelling C, Hortobagyi GN (2001). Paclitaxel in the multimodality treatment for inflammatory breast carcinoma. Cancer.

[31] Takahashi T, Akashi-Tanaka S, Fukutomi T, Watanabe T, Katsumata N, Miyakawa K, Hasegawa T, Tsuda H (2001). Two special types of breast cancer presenting as progressive disease after neoadjuvant chemotherapy with docetaxel plus doxorubicin. Breast Cancer.

[32] Leopold PL, Vincent J, Wang H (2012). A comparison of epithelial-to-mesenchymal transition and re-epithelialization. Semin Cancer Biol.

[33] Rosen EM, Pishvaian MJ (2014). Targeting the BRCA1/2 tumor suppressors. Curr Drug Targets.

[34] Khoury-Haddad H, Guttmann-Raviv N, Ipenberg I, Huggins D, Jeyasekharan AD, Ayoub N (2014). PARP1-dependent recruitment of KDM4D histone demethylase to DNA damage sites promotes double-strand break repair. Proc Natl Acad Sci USA.

[35] Węsierska-Gądek J, Zulehner N, Ferk F, Składanowski A, Komina O, Maurer M (2012). PARP inhibition potentiates the cytotoxic activity of C-1305, a selective inhibitor of topoisomerase II, in human BRCA1-positive breast cancer cells. Biochem Pharmacol.

[36] Shen Y, Rehman FL, Feng Y, Boshuizen J, Bajrami I, Elliott R, Wang B, Lord CJ, Post LE, Ashworth A (2013). BMN 673, a novel and highly potent PARP1/2 inhibitor for the treatment of human cancers with DNA repair deficiency. Clin Cancer Res.

[37] Fojo T, Bates S (2013). Mechanisms of resistance to PARP inhibitors - three and counting. Cancer Discov.

[38] Yin S, Xu L, Bandyopadhyay S, Sethi S, Reddy KB (2011). Cisplatin and TRAIL enhance breast cancer stem cell death. Int J Oncol.

[39] Potemski P, Kusińska R, Pasz-Walczak G, Piekarski JH, Watała C, Płuciennik E, Bednarek AK, Kordek R (2009). Prognostic relevance of cyclin E expression in operable breast cancer. Med Sci Monit.

[40] Willmarth NE, Albertson DG, Ethier SP (2004). Chromosomal instability and lack of cyclin E regulation in hCdc4 mutant human breast cancer cells. Breast Cancer Res.

